# Predictors of cognitive, behavioural and academic difficulties in NF1

**DOI:** 10.1192/bjo.2021.649

**Published:** 2021-06-18

**Authors:** Kavitha Chinnappa Ramamurthy, Marie-Maude Geoffray, Louise Robinson, Lauren Manderson, Julieta O'Flaherty, Annukka Lehtonen, Grace Vassallo, Shruti Garg, Jonathan Green

**Affiliations:** 1 Department of Child and Adolescent Psychiatry ,Manchester University Foundation Trust; 2 Centre hospitalier le Vinatier; 3 Manchester Centre for Genomic Medicine, Manchester University NHS Foundation Trust, Manchester Academic Health Sciences Centre, Department of Child and Adolescent Psychiatry, Manchester University Foundation Trust; 4 Division of Neuroscience and Experimental Psychology, Faculty of Biological Medical & Health Sciences, University of Manchester, Manchester Academic Health Sciences Centre, Department of Child and Adolescent Psychiatry, Manchester University Foundation Trust

## Abstract

**Aims:**

The aim of this study is to systematically investigate the demographic and disease predictors of cognitive and behavioural phenotype in the largest cohort of children with NF1 published to date. Based on previously published research, we examine the potential role of demographic predictors such as age, sex, SES, parental NF1 status as well as the neurological complications such as epilepsy and brain tumours in NF1 associated cognitive/ behavioural impairments.

**Method:**

In this cross-sectional study design, participant data were drawn from two large databases which included (i) A clinical database of all patients with NF1 seen in a clinical psychological service from 2010 to 2019 and (ii) A research dataset from two previously published studies (2,8). The complex National NF1 service based within Manchester regional genetic services is set up for individuals with complex NF1 (https://www.mangen.co.uk/healthcare-professionals/clinical-genomic-services/nf1/) in the North of the UK. Children were referred to the psychological services by NF1 clinicians if psychological assessment was warranted based on parental reports. In order to reduce clinic referral bias, the clinical sample was supplemented by including participants that were seen solely for the purposes of research studies within our centre.

**Result:**

Relative to population norms, 90% of the NF1 sample demonstrated significantly lower scores in at least one cognitive or behavioral domain. Family history of NF1 and lower SES were independently associated with poorer cognitive, behavioral and academic outcomes. Neurological problems such as epilepsy and hydrocephalus were associated with lower IQ and academic skills.

**Conclusion:**

Cognitive and behavioural phenotypes commonly emerge via a complex interplay between genes and environmental factors, and this is true also of a monogenic condition such as NF1. Early interventions and remedial education may be targeted to risk groups such those with familial NF1, families with lower SES and those with associated neurological comorbidities.

